# Respiratory distress observation scales to predict weaning outcome

**DOI:** 10.1186/s13054-022-04028-7

**Published:** 2022-06-06

**Authors:** Maxens Decavèle, Emmanuel Rozenberg, Marie-Cécile Niérat, Julien Mayaux, Elise Morawiec, Capucine Morélot-Panzini, Thomas Similowski, Alexandre Demoule, Martin Dres

**Affiliations:** 1 INSERM, UMRS1158 Neurophysiologie Respiratoire Expérimentale et Clinique, Sorbonne Université, 75005 Paris, France; 2 APHP, Groupe Hospitalier Universitaire APHP-Sorbonne Université, site Pitié-Salpêtrière, Service Médecine Intensive et Réanimation (Département R3S), 75013 Paris, France; 3 APHP, Groupe Hospitalier Universitaire APHP-Sorbonne Université, site Pitié-Salpêtrière, Service de Pneumologie (Département R3S), 75013 Paris, France; 4 APHP, Groupe Hospitalier Universitaire APHP-Sorbonne Université, site Pitié-Salpêtrière, Département R3S, 75013 Paris, France

**Keywords:** Critical care, Dyspnea, Dyspnea observation scale, Respiratory Distress Observation Scale, Intensive care unit, Spontaneous breathing trial, Weaning from mechanical ventilation

## Abstract

**Background:**

Whether dyspnea is present before starting a spontaneous breathing trial (SBT) and whether it may affect the outcome of the SBT is unknown. Mechanical Ventilation—Respiratory Distress Observation Scale (MV-RDOS) has been proposed as a reliable surrogate of dyspnea in non-communicative intubated patients. In the present study, we sought (1) to describe the evolution of the MV-RDOS during a SBT and (2) to investigate whether MV-RDOS can predict the outcome of the SBT.

**Methods:**

Prospective, single-center study in a twenty-two bed ICU in a tertiary center. Patients intubated since more 48 h who had failed a first SBT were eligible if they meet classical readiness to wean criteria. The MV-RDOS was assessed before, at 2-min, 15-min and 30-min (end) of the SBT. The presence of clinically important dyspnea was inferred by a MV-RDOS value ≥  2.6.

**Results:**

Fifty-eight patients (age 63 [51–70], SAPS II 66 [51–76]; med [IQR]) were included. Thirty-three (57%) patients failed the SBT, whose 18 (55%) failed before 15-min. Twenty-five (43%) patients successfully passed the SBT. A MV-RDOS ≥ 2.6 was present in ten (17%) patients before to start the SBT. All these ten patients subsequently failed the SBT. A MV-RDOS ≥ 2.6 at 2-min predicted a SBT failure with a 51% sensibility and a 88% specificity (AUC 0.741 95% confidence interval [CI] 0.616–0.866, *p* = 0.002). Best cut-off value at 2-min was 4.3 and predicted SBT failure with a 27% sensibility and a 96% specificity.

**Conclusion:**

Despite patients met classical readiness to wean criteria, respiratory distress assessed with the MV-RDOS was frequent at the beginning of SBT. Measuring MV-RDOS before to initiate a SBT could avoid undue procedure and reduce patient’s exposure to unnecessary mechanical ventilation weaning failure and distress.

**Supplementary Information:**

The online version contains supplementary material available at 10.1186/s13054-022-04028-7.

## Background

The decision to extubate poses critical challenges. Delaying extubation exposes patients to undue prolongation of mechanical ventilation [1] and the ensuing complications whereas extubation failure increases morbi-mortality [2]. The decision to extubate comes after a patient has been considered "ready to wean" [1] and at the end of a spontaneous breathing trial (SBT) mimicking the post-extubation load-capacity balance of the respiratory system.

Judging readiness to wean and SBT outcome are difficult, as demonstrated by discrepancies in clinicians’ assessments [3, 4] and high incidences of SBT and extubation failure [3–5]. To diagnose SBT failure, international recommendations suggest relying on objective criteria such as respiratory rate, heart rate or arterial blood gases. However, it is well established that the separation from the ventilator is often based on subjective grounds, with a frequent natural tendency for clinicians to keep their patients on the "safe" side, i.e., considering them as not being ready for separation [6]. Only very few studies have investigated the patients' subjective perception of autonomous breathing [7]. This is surprising since dyspnea, a cardinal symptom of respiratory distress may behave as a warning signal suggesting that patients are not ready to undergo a SBT.

Dyspnea corresponds to the self-report of a bothering or distressing awareness of breathing activity [8]. It is considered to result from an imbalance between the outflowing neural drive to breathe—the "demand," from the brain—and the corresponding instreaming afferent return—the "supply," from the respiratory system—(corollary discharge theory) [9]. This can typically result from an unfavorable respiratory system load-capacity balance, as it occurs in cases of SBT failure [10]. The association between dyspnea and SBT failure has indeed been reported [11–15], as well as post-extubation dyspnea is predictive of extubation failure [16].

Yet, assessing dyspnea in intubated mechanically ventilated patients is not straightforward. Self-report implies patient cooperation, which is often not possible in this setting [17]. Subjective nonself evaluation of dyspnea is unreliable [11, 18, 19]. In addition, dyspnea is multidimensional in essence, a characteristic not captured by the numerical rating scales typically used in the clinical field [8]. Respiratory distress observation scales (RDOS) were developed to obviate these limitations, in palliative care medicine [20], upon admission to the intensive care unit (IC-RDOS [21]) or under mechanical ventilation (MV-RDOS [22]). These scales rely on assessing a multidimensional ensemble of measurable physical and behavioral manifestations related to respiratory suffering [18], correlate with dyspnea in communicative patients [20–22] and provided standardized way to infer dyspnea in noncommunicative patients.

The present study was designed to test the hypothesis that MV-RDOS would be valuable to refine readiness to wean criteria and predict SBT failure. The objective was to measure MV-RDOS before starting the SBTs in patients meeting readiness to wean criteria, and to repeat this evaluation at 2 min-SBT, 15 min-SBT and 30 min-SBT (SBT end). We confronted the corresponding measurements with SBT outcomes.

## Patients and methods

### Study design and settings

We assessed unpublished data from a previous a single-center prospective weaning study [23] that was approved by the Comité de Protection des Personnes du Sud Ouest et Outre Mer 4 (RCB ID: 2018-A00176-49). Written and oral information about the study was given to patients or their families prior enrolment. Informed consent was obtained from all patients or their relatives. The present reporting complies with the Strengthening the Reporting of Observational Studies in Epidemiology (STROBE) Statement.

Patients were eligible for participation if they met all the following criteria: (1) mechanical ventilation via an endotracheal tube for more than 48 h, (2) failure to a first SBT, (3) readiness-to-wean criteria on the day of inclusion defined as follows: (1) adequate motor responses to simple verbal commands, (2) SpO_2_ > 90% or PaO_2_/FiO_2_ ≥ 150 mmHg with a fraction of inspired oxygen (FiO_2_) ≤ 40% and (3) positive end-expiratory pressure ≤ 8 cmH_2_O (4) heart rate < 140 beats/min and respiratory rate < 35 cycles/min [1]. Patients younger than 18, pregnant women, and patients in whom weaning was impossible (pre-existing neuromuscular disorders, cervical cord injury) were not considered for inclusion.

### Data collection and respiratory distress assessment

Physiological variables such as respiratory rate, heart rate, systolic blood pressure, SpO_2_ and Glasgow coma scale were recorded before and at the end of the SBT. Arterial blood gas analyses were sampled before and at the end of the SBT according to local practices.

A single investigator E.R evaluated in real time the MV-RDOS (see below) and the Rapid shallow breathing index (RSBI). The MV-RDOS is a 5-items "respiratory distress observation scale" specifically designed for and validated in mechanically ventilated patients. MV-RDOS items comprise respiratory rate, use of neck muscles during inspiration, inward abdominal motion during inspiration (abdominal paradox), heart rate, and facial expression of fear. MV-RDOS is strictly clinical, standardized, and does not require patient cooperation. In communicative ICU patients, a MV-RDOS of 2.6 predicts a dyspnea visual analog scale > 30 mm with a 57% sensitivity, a 94% specificity, and an AUC of 0.782 (95% CI 0.581–0.982) [22], noting that a dyspnea visual analog scale (VAS) > 30 mm is considered clinically important [24], MV-RDOS was only gathered by the investigator for research purpose and not for clinical decision-making. The MV-RDOS was thus presented using its raw values and was also dichotomized around its 2.6 threshold value that corresponds to a high probability of clinically important self-reported dyspnea.

The RSBI defined by the respiratory rate/tidal volume ratio (*fR*/*V*T) was continuously obtained from the ventilator.

### Study design

Patients enrolled in the study underwent a 30-min SBT (shorter in case of obvious clinical intolerance) with pressure support and positive end-expiratory pressure set to zero, while FiO_2_ remained unchanged [25, 26]. SBT failure was defined by the occurrence of at least one of the following objective criteria: respiratory rate  ≥ 35 breaths/min or increase  ≥ 50% from baseline, SpO_2_ ≤ 90% or PaO_2_ ≤ 50 mmHg with FiO_2_  ≥ 50%), PaCO_2_ > 50 mmHg, heart rate ≥ 140 bpm, de novo supraventricular or ventricular arrhythmia, systolic arterial pressure > 180 or < 90 mmHg, or alteration of consciousness [1].

After obtaining consent and before starting the SBT, MV-RDOS and the rapid shallow breathing index (RSBI, *fR*/*V*T) were collected. Then, the SBT started. MV-RDOS and RSBI were then evaluated at the second minute, at the fifteenth minute and at the end of the SBT or earlier in case of failure.

### Statistical analysis

Continuous variables were expressed as median (interquartile range) and categorical variables were expressed as absolute and relative frequencies. Continuous variables were analyzed by Mann–Whitney test and categorical variables were compared using the Chi2-test or Fisher's exact test depending on the number of categories per variable. The MV-RDOS and RSBI values before, at 2-, 15- and 30-min of SBT were compared using a nonparametric analysis of variance test followed by Dunn’s multiple comparison test. The performance of the MV-RDOS to discriminate SBT failure and success was tested before, at 2- and at 15-min of SBT by generating receiver operating curves (ROC), which were compared to the ROC of the RSBI. At same time points, sensitivity and specificity were calculated for the MV-RDOS cut-off value of 2.6 (representing clinically important self-reported dyspnea) as well as the best MV-RDOS and RSBI sensitivity and specificity according to the highest likelihood ratio. Analyses were performed using Prism 9.3.0 software (GraphPad Software, USA).

## Results

### Characteristics of the patients and SBT outcome

During the study period, 794 patients were admitted to the ICU and 340 received invasive mechanical ventilation. Among these 340 patients, 60 were enrolled in the study but 58 were analyzed because of missing data in the MV-RDOS calculation in two patients.

**Fig. 1:**
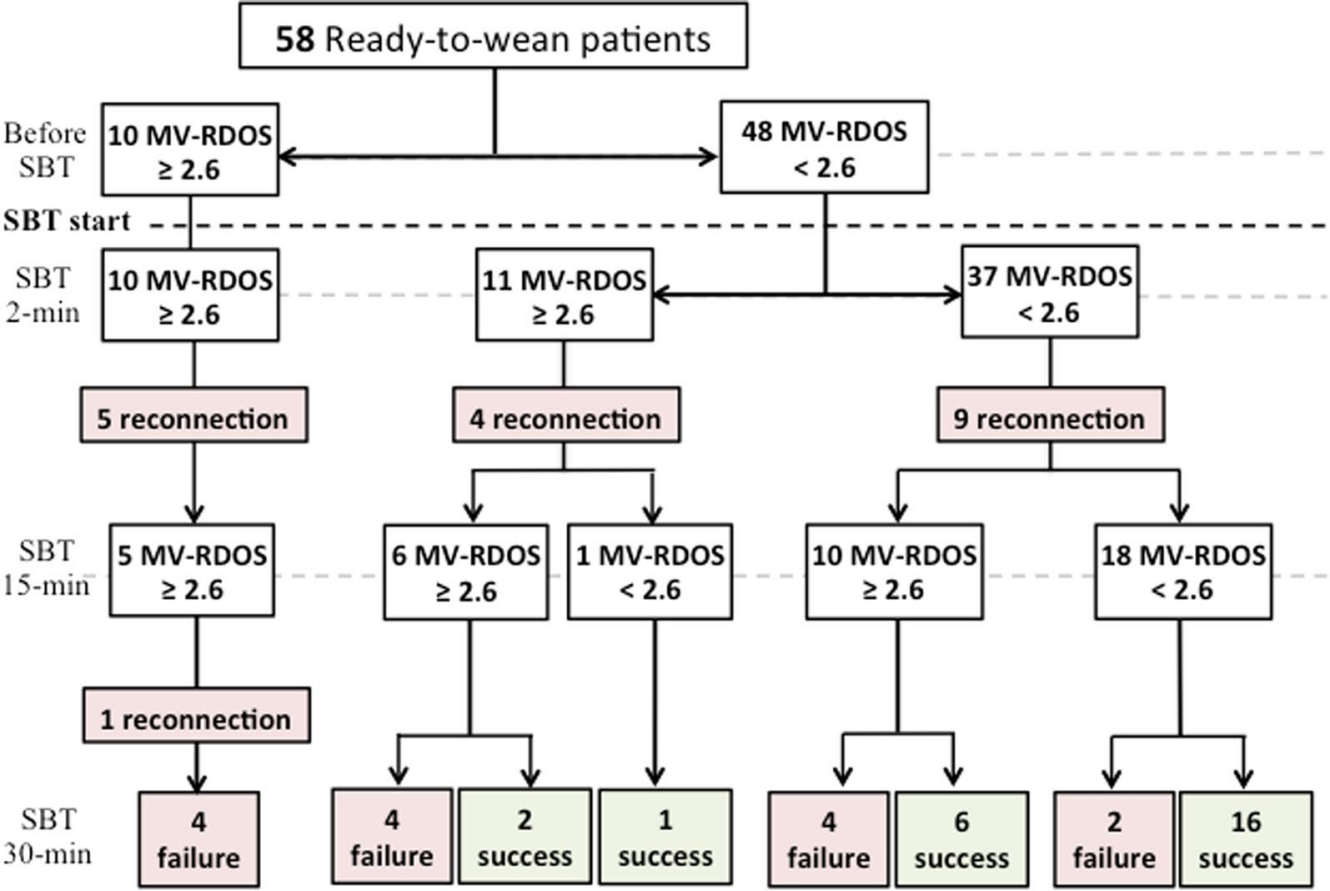
Number of patients with spontaneous breathing trial (SBT) failure and success according to the presence or absence of a Mechanical Ventilation—Respiratory Distress Observation Scale (MV-RDOS) > 2.6

Table 1 presents the main characteristics of the patients. Thirty-three (57%) of the 58 patients enrolled in the study failed the SBT (Fig. 1). Among these 33 patients who failed the SBT, 18 (55%) failed before 15-min, 1 (3%) failed between 15- and 30-min and 14 (42%) failed at 30-min (Fig. 1). Except a lower PaO_2_/FiO_2_ ratio and a higher proportion of cardiac arrest as reason for intubation in patients who succeed, no difference was found among characteristics at inclusion between patients who succeed the SBT and their counterparts (Table 1). Details on the distribution of SBT failure criteria are reported in Additional file 1: Table S1.

**Table 1:** Characteristics of the patients

Variables	All patients (*n* = 58)	SBT success (*n* = 25)	SBT failure (*n* = 33)	*P* value
Age, years	63 (51–68)	62 (50–73)	63 (52–68)	0.922
Gender (male), *n* (%)	31 (53)	15 (60)	16 (48)	0.384
Body mass index, kg/m^2^	25 (21–29)	25 (20–31)	24 (21–29)	0.782
Comorbidities				
Arterial hypertension, *n* (%)	21 (36)	9 (36)	12 (36)	1.000
COPD, *n* (%)	11 (19)	5 (20)	6 (18)	1.000
Diabetes, *n* (%)	11 (19)	4 (16)	7 (21)	0.742
Chronic kidney failure	9 (16)	5 (20)	4 (12)	0.479
Reasons for ICU admission				
Acute respiratory failure, *n* (%)	33 (57)	15 (60)	18 (54)	0.678
Coma, *n* (%)	13 (22)	6 (24)	7 (21)	1.000
Cardiac arrest, *n* (%)	8 (14)	2 (8)	6 (18)	0.001
Other, *n* (%)	4 (7)	2 (8)	2 (6)	1.000
Clinical variables				
Length of MV, days (at inclusion)	5 (2–7)	3 (2–6)	5 (2–11)	0.315
Number of previous SBT (at inclusion)	1 (1–1)	1 (1–1)	1 (1–1)	0.731
SAPS II (at admission)	63 (49–76)	57 (46–75)	66 (50–76)	0.388
SOFA (at admission)	10 (7–12)	10 (7–11)	10 (7–12)	0.591
Readiness to wean assessment at inclusion				
Heart rate, beats/min	93 (84–100)	92 (79–101)	93 (84–99)	0.735
Systolic arterial blood pressure, mmHg	132 (116–145)	125 (111–141)	140 (117–152)	0.094
Bicarbonate, mmol/L	27 (23–31)	25 (19–29)	28 (24–32)	0.105
PaO_2_/FiO_2_	248 (178–329)	315 (211–355)	230 (157–285)	0.014
Positive end-expiratory pressure, cmH_2_O	5 (5–6)	5 (5–6)	6 (5–6)	0.092
Respiratory rate, breaths/min	22 (17–27)	19 (16–25)	22 (17–29)	0.189
Expired tidal volume, ml/IBW	6.9 (6.2–8.4)	7.0 (6.6–9.2)	6.8 (5.8–8.2)	0.167

COPD, chronic obstructive pulmonary disease; *fR*, respiratory rate; *V*T, expired tidal volume; SBT, spontaneous breathing trial; SAPS II, Simplified Acute Physiology Score (SAPS) II; SOFA, Sequential Organ Failure Assessment

### Respiratory distress assessment before starting the spontaneous breathing trial

Before the SBT, the MV-RDOS and RSBI were, respectively, 2.2 (2.0–2.3) and 47 (34–66). The MV-RDOS was 2.1 (2.0–2.2) in patients who succeeded and 2.3 (2.0–2.6) in those who failed (*p* = 0.014). A MV-RDOS  ≥ 2.6 was present in ten patients (17%) before starting the SBT. All these patients subsequently failed the SBT.

The RSBI was also significantly lower in patients who succeeded than in the counterparts (37 [29–54] vs. 56 (39–73), *p* = 0.010).

### MV-RDOS assessment over the spontaneous breathing trial

At 2-min after the beginning of the SBT, 21 (36%) patients had a MV-RDOS  ≥ 2.6 although no objective SBT failure criteria were present. Among these 21 patients, 18 (86%) subsequently failed the SBT. At 15-min of SBT, in 21 (53%) patients, the MV-RDOS was higher to 2.6 while no objective SBT failure criteria were present. Among these 21 patients, 12 (57%) subsequently failed the SBT. At 30-min of SBT (end), 22 (56%) patients had a MV-RDOS  ≥ 2.6 without presenting objective SBT failure criteria. Among these 22 patients, 12 (55%) failed the SBT (Fig. 1).

**Fig. 2:**
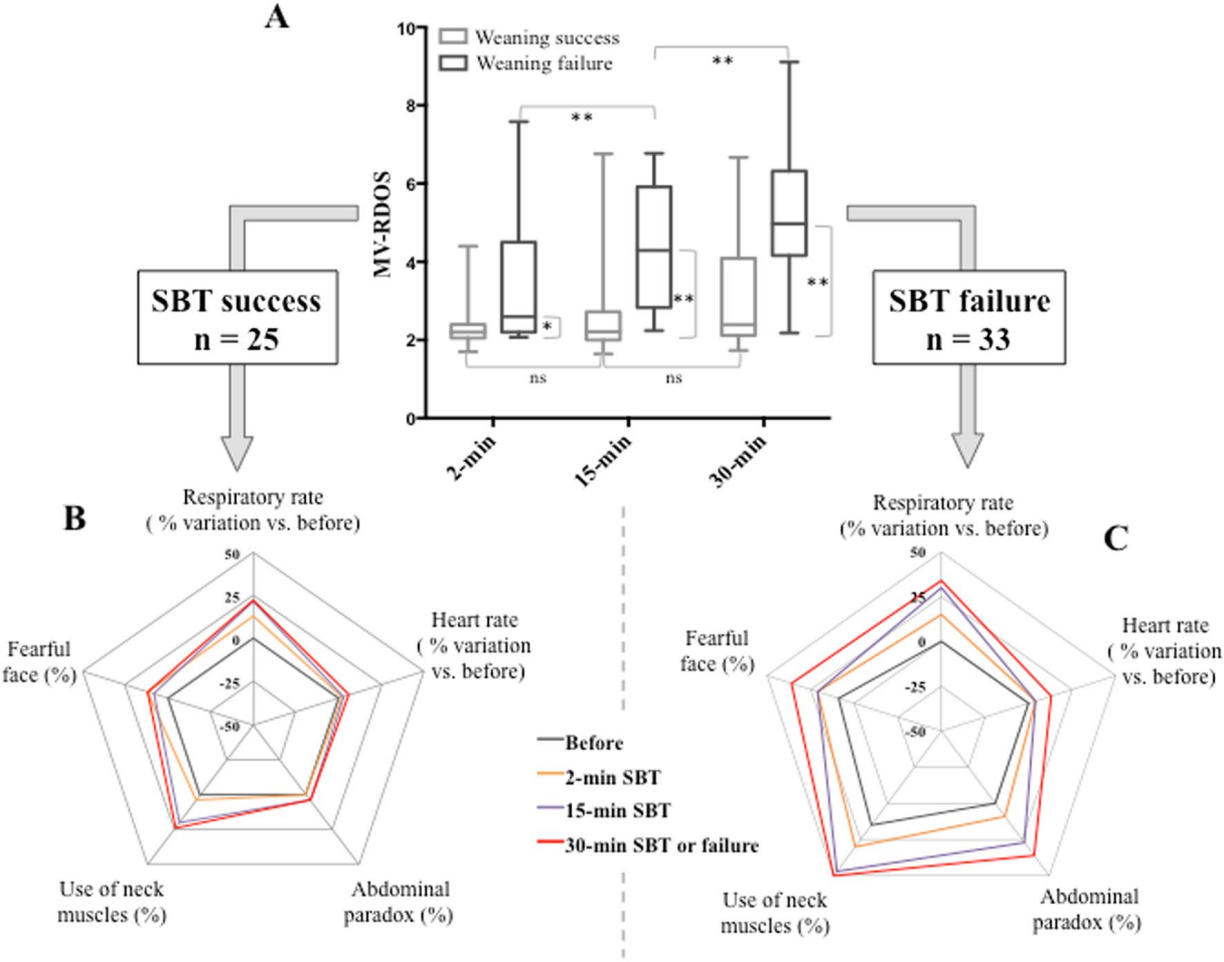
Evolution of the Mechanical Ventilation—Respiratory Distress Observation Scale (MV-RDOS, Panel A) and all its components during the spontaneous breathing trial (SBT) between patients who succeeded (Panel B) or failed (Panel C) the SBT. *p < 0.05, **p < 0.001

The MV-RDOS significantly increased during the SBT in patients who failed (*p* < 0.001) whereas it did not significantly vary in patients who successfully passed (*p* = 0.831) the SBT (Fig. 2). MV-RDOS and RBSI values over the SBT are available in Table 2 and Additional file 1: Table S2.

**Table 2:** Respiratory distress assessment over the spontaneous breathing trial (SBT) between patients who succeeded or failed the SBT

Variables of respiratory distress assessment	All patients (*n* = 58)	SBT success (*n* = 25)	SBT failure (*n* = 33)	*P* value
*Before SBT (n = 58)*				
*fR*/*V*T, breaths/min/L	47 (34–66)	37 (29–54)	56 (39–73)	0.010
MV-RDOS ≥ 2.6, *n* (%)	10 (17)	0 (0)	10 (24)	0.008
MV-RDOS value	2.2 (2.0–2.3)	2.1 (2.0–2.2)	2.3 (2.0–2.6)	0.014
Heart rate, beats/min	92 (84–100)	91 (84–98)	94 (84–101)	0.410
Respiratory rate, cycles/min	22 (17–27)	19 (14–23)	22 (18–28)	0.027
Use of neck muscle during inspiration*, n* (%)	5 (9)	0 (0)	5 (15)	0.063
Abdominal paradox during inspiration*, n* (%)	0 (0)	0 (0)	0 (0)	1.000
Facial expression of fear*, n* (%)	3 (5)	0 (0)	3 (9)	0.251
*At 2-min SBT (n = 58)*				
*fR*/*V*T, *breaths/min/L*	64 (49–79)	52 (41–72)	68 (57–88)	0.010
MV-RDOS ≥ 2.6, *n* (%)	21 (38)	3 (12)	18 (55)	< 0.001
MV-RDOS value	2.3 (2.2–4.2)	2.2 (2.1–2.4)	2.6 (2.2–4.5)	0.003
Heart rate, beats/min	96 (88–102)	92 (86–102)	99 (90–104)	0.063
Respiratory rate*, *cycles/min	25 (20–30)	23 (20–26)	28 (24–32)	0.005
Use of neck muscle during inspiration*, n* (%)	11 (19)	1 (4)	10 (30)	0.016
Abdominal paradox during inspiration*, n* (%)	3 (5)	0 (0)	3 (9)	0.251
Facial expression of fear*, n* (%)	10 (17)	3 (12)	7 (21)	0.489
*At 15-min SBT (n = 40)*			*n* = 15	
*fR*/*V*T, breaths/min/L	67 (45–89)	50 (40–72)	93 (60–141)	0.002
MV-RDOS ≥ 2.6, *n* (%)	21 (53)	8 (32)	13 (87)	< 0.001
MV-RDOS value	2.7 (2.2–4.3)	2.3 (2.1–4.1)	4.3 (2.8–5.3)	< 0.001
Heart rate, beats/min	96 (86–102)	92 (82–101)	97 (88–109)	0.492
Respiratory rate, cycles/min	25 (21–31)	22 (18–26)	31 (27–34)	0.004
Use of neck muscle during inspiration*, n* (%)	12 (30)	5 (20)	7 (47)	0.091
Abdominal paradox during inspiration*, n* (%)	5 (13)	1 (4)	4 (27)	0.056
Facial expression of fear*, n* (%)	5 (13)	2 (8)	3 (20)	0.345
*At 30-min SBT (n = 39)*			*n* = 14	
*fR*/*V*T, breaths/min/L	72 (49–107)	56 (41–78)	94 (60–141)	0.006
MV-RDOS ≥ 2.6, *n* (%)	22 (56)	10 (40)	12 (86)	< 0.001
MV-RDOS value	3.9 (2.2–4.5)	2.4 (2.1–4.2)	4.8 (4.1–6.2)	< 0.001
Heart rate, beats/min	98 (89–105)	93 (87–103)	101 (98–116)	0.061
Respiratory rate, cycles/min	27 (22–32)	24 (21–28)	33 (27–38)	0.007
Use of neck muscle during inspiration*, n* (%)	13 (33)	6 (24)	7 (50)	0.157
Abdominal paradox during inspiration*, n* (%)	6 (15)	1 (4)	5 (36)	0.016
Facial expression of fear*, n* (%)	8 (21)	3 (12)	5 (36)	0.108

MV-RDOS, mechanical ventilation—respiratory distress observation scale; *f*R, respiratory rate; VT, expired tidal volume

### Prediction of SBT outcome according to MV-RDOS values

**Fig. 3:**
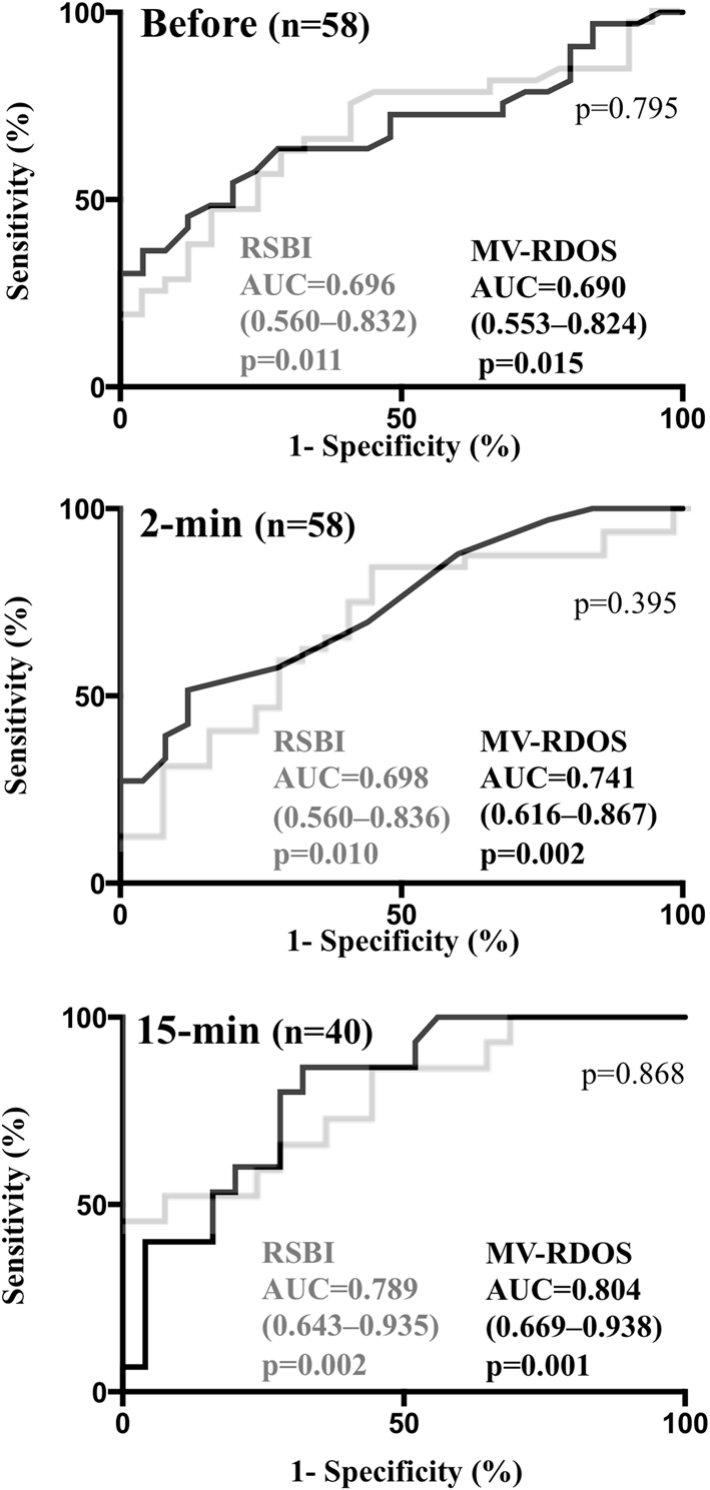
Performance of the Mechanical Ventilation—Respiratory Distress Observation Scale (MV-RDOS, black) and the rapid shallow breathing index (RSBI, grey) recorded before, at 2-min and at 15-min of a spontaneous breathing trial (SBT) to predict SBT failure. AUC—area under receiver operating curve

Figure 3 represents the performances of the MV-RDOS to predict the SBT failure, at different time points of the SBT. A MV-RDOS  ≥ 2.6 before SBT predicted a SBT failure with a 30% sensibility and a 100% specificity (AUC 0.690 95% confidence interval [CI] 0.553–0.824, *p* = 0.015). Best cut-off value (highest likelihood ratio) before SBT was 2.4 and predicted SBT failure with a 36% sensibility and a 96% specificity.

A MV-RDOS of 2.6 at 2-min predicted a SBT failure with a 51% sensibility and a 88% specificity (AUC 0.741 95% confidence interval [CI] 0.616–0.866, *p* = 0.002). Best cut-off value at 2-min was 4.3 and predicted SBT failure with a 27% sensibility and a 96% specificity.

A MV-RDOS of 2.6 at 15-min predicted a SBT failure with a 87% sensibility and a 68% specificity (AUC 0.783 95%CI 0.642–0.924, *p* = 0.003). Best cut-off value at 15-min was 4.5 and predicted SBT failure with a 40% sensibility and a 96% specificity.

At each time points, the MV-RDOS performance to predict a SBT failure was similar to that of the RSBI (Fig. 3). Before SBT, best cut-off value of RSBI was 71 and predicted a SBT failure with a 27% sensibility and a 96% specificity. At 2-min, best cut-off value of RSBI was 84 and predicted a SBT failure with a 33% sensibility and a 92% specificity. At 15-min best cut-off value of RSBI was 96 and predicted a SBT failure with a 46% sensibility and a 96% specificity.

## Discussion

The main findings can be summarized as follows: (1) almost 20% of patients, yet clinically deemed ready to undergo a SBT, had a MV-RDOS  ≥ 2.6 suggesting a high probability of clinically important dyspnea (VAS > 30 mm), (2) 100% of these patients with a MV-RDOS ≥ 2.6 before SBT subsequently failed the SBT, (3) an early MV-RDOS assessment at 2-min and 15-min SBT could predict SBT failure with a high specificity.

### Prevalence and significance of respiratory distress before SBT

Observational studies of intubated patients in the ICU setting show a 40% prevalence of self-reported dyspnea (5 [4–7] on a 0–10 VAS) on the first day on which patient were able to communicate [27, 28]. The inferred prevalence of clinically important dyspnea (MV-RDOS ≥ 2.6) observed in our study is roughly two-fold lesser (17%). This could be explained by the fact in our study, patients were enrolled at a more clinically stable stage of their disease and were not yet facing the SBT asphyxial threat. Indeed, at the end of the SBT, clinically important dyspnea was finally suspected (MV-RDOS ≥ 2.6) in 56% of patients, which is in line with the previously reported proportion of patient with self-reported dyspnea ≥ 4 at the end of a 30-min SBT (62%) [11].

Our study showed that the MV-RDOS provides a standardized assessment of respiratory distress before SBT which, once detected, allowed to identify patients with a particularly high risk of SBT failure. To the best of our knowledge, this is the first study that evaluate the performance of a respiratory distress assessment to predict the SBT outcome. Although two items of the MV-RDOS, the heart and breathing rate, are present in the readiness-to-wean current criteria, only their threshold values are retained in the guidelines (i.e., heart rate ≥ 140 beats/min, respiratory rate ≥ 35 cycles/min). Here, the MV-RDOS allowed to integrate all absolute values of the heart and respiratory rate irrespective of any threshold values.

### Evolution of respiratory distress across the SBT

In our study the MV-RDOS dramatically increased over the SBT in patients who failed the SBT. The asphyxial threat induced by the SBT, especially in those who failed, induce an innate array of multidimensional behaviors that have previously been investigated [29–31]. Schematically, these include signs of autonomic system activation (heart rate), respiratory drive increase (respiratory rate, abdominal paradox, use of neck muscle) or emotional response (facial expression of fear). This two-fold increase in MV-RDOS at 15-min SBT in patients who failed the SBT is in line with the two-fold increase observed in the RDOS at 15-min SBT in patients with terminal ventilator withdrawal [30]. In another study, including patient who already presented sign of respiratory distress during a previous SBT, the use of neck muscle, the abdominal paradox and a fearful facial expression were observed during the second SBT in 58%, 33% and 58% of cases, respectively. These incidences were similar to that observed in our study except for the fearful facial expression which was less frequently observed in our study. This last point could be explained, at least partially, by the observer/investigator subjectivity or empathy variability between studies [18, 32]. All these three manifestations of respiratory suffering generally appeared before the first five minutes of SBT [29, 30], as observed in our study, supporting the relevance of using the MV-RDOS even at very early SBT stage. A physiological study reported that neck muscle electromyographic activity significantly increased during the first minute of patients who failed the SBT [33]. In addition, all the 5 items of the MV-RDOS significantly increased across the SBT, supporting also the clinical relevance of each separate item of the scale.

### Clinical implications

According to guidelines, the assessment of readiness to wean, a crucial step in the weaning process, relies on a checklist of objective criteria [1]. This is however surprising that patient’s dyspnea is not taking into account when deciding to initiate a SBT. Our study shows that a significant proportion of patients (almost 20%) exhibited MV-RDOS ≥ 2.6 (suggesting clinically important dyspnea) before starting the SBT. It is also a major result that all these patients subsequently failed the SBT suggesting that they were exposed to an unnecessary respiratory suffering [17] recently demonstrated to be associated with the occurrence of post-traumatic stress disorder [28]. This lack of recognition of dyspnea is probably explained by the difficulty to obtain a reliable assessment of patient’s dyspnea in the ICU [28]. Using hetero-evaluation scales may help to address this issue. Systematic assessment of respiratory distress using the MV-RDOS before the SBT may help to refine the readiness-to-wean criteria. A MV-RDOS value ≥ 2.6 could be used to not initiate or promptly stop the SBT because corresponds to clinically important dyspnea and suffering. In the case of pain, an intensity rating ≥ 4 is also the lower cut-off for “moderate-to-severe pain” and constitutes a clear indication for prompt analgesic prescription [34] and it seems obvious to stop muscle rehabilitation in case of pain during exercise. The particularly high specificity of MV-RDOS value > 4 (accompanied with modest sensitivity) may be helpful for clinical decision making regarding this goal of care of minimizing traumatic experiences induced by respiratory suffering [35] even more as less demanding weaning strategies are not necessarily associated with lower rates of successful weaning [36].

### Limitations

This study has several limitations. Firstly, self-reported dyspnea was not measured, and it would have been interesting to confront dyspnea and MV-RDOS performances to predict SBT outcomes. However, MV-RDOS strongly correlates with dyspnea [22] and contrarily to self-reported dyspnea (unidimensional assessment of dyspnea intensity by numerical rating scales), MV-RDOS integrates the multiple dimensions of respiratory suffering and could be reach in every patient irrespective of their self-report capabilities. Secondly, this study was conducted exclusively in patients who already failed at least one SBT since these patients represented a priori greater clinical challenge. This limits the generalizability of our results and further studies are warranted in patients who never attempt yet a SBT. Thirdly, RSBI median values in our study were lower than that reported in the princeps study by Tobin et al., but in this study simple-to-wean patients were included, RSBI predicted extubation failure (not SBT failure) and currently median RSBI value in patients who failed the SBT is around 85 [37]. Fourthly, although MV-RDOS provide standardization in clinical assessment of respiratory distress, assessment of facial expression of fear and paradoxical motion of the abdomen during inspiration may vary between observers. Inter-rater reliability has not been assessed in this exploratory study. Finally, RSBI provided also good performances to predict SBT outcomes and contrarily to the MV-RDOS its assessment is entirely objective. However, beyond the prediction of the SBT outcome, the MV-RDOS allows to identify or strongly suspect a major patient-centered outcome—dyspnea—and when appropriate, to treat it. Conversely, and to the best of our knowledge, RSBI values has never been proposed to infer clinically important dyspnea in critically ill patients receiving invasive mechanical ventilation.

## Conclusion

MV-RDOS, an observational clinical corollary to dyspnea might be useful to refine readiness-to-wean criteria. In patient who entered in a SBT, the MV-RDOS might be useful also for early SBT discontinuation in those who will certainly failed the SBT, minimizing exposition to unnecessary respiratory suffering and its associated burden. Such clinical approach may be integrated in a more general goal of care centered on patient comfort and limiting traumatic experience of the ICU stay [28]. Performance and inter-rater reliability of the MV-RDOS in predicting SBT outcome should be confirmed in multicenter study.

## Supplementary Information

**Additional file 1.** Spontaneous breathing trial (SBT) failure criteria and Respiratory distress assessment over the spontaneous breathing trial (SBT).

## Data Availability

The datasets analyzed during the current study are available from the corresponding author on reasonable request.

## Abbreviations

AUC: Area under the curve
CI: Confidence interval
COPD: Chronic obstructive pulmonary disease
*f*R: Respiratory rate
ICU: Intensive care unit
MV-RDOS: Mechanical Ventilation—Respiratory Distress Observation Scale
OR: Odds ratio
ROC: Receiver operating curve
RSBI: Rapid shallow breathing index
SAPS II: Simplified acute physiology score II
SBT: Spontaneous breathing trial
SOFA: Sequential Organ Failure Assessment
VT: Expired tidal volume
