# Supplementary material for: Respiratory distress observation scales to predict weaning outcome
Source: Crit Care. 2022 Jun 6;26:162. doi: 10.1186/s13054-022-04028-7 (PMC9169318; doi:10.1186/s13054-022-04028-7)
Supplement: Supplementary file 1 — Additional file 1. Spontaneous breathing trial (SBT) failure criteria and Respiratory distress assessment over the spontaneous breathing trial (SBT). [file 13054_2022_4028_MOESM1_ESM.docx]

**Additional file 1:**

**"Respiratory distress observation scales to predict weaning outcome"**

Maxens Decavèle MD^1,2^, Emmanuel Rozenberg M.D.^2^, Marie-Cécile Niérat, PhD^2^,

Julien Mayaux, MD^2^, Elise Morawiec, MD^2^, Capucine Morélot-Panzini MD, PhD^1,3^,

Thomas Similowski MD, PhD^1,4^, Alexandre Demoule MD, PhD^1,2^, Martin Dres MD, PhD^1,2^

**Supplemental Digital Content**

**Table S1. Numbers and proportions of spontaneous breathing trial (SBT) failure criteria identified in the 33 patients who failed the SBT.**

**Table S2. Respiratory distress assessment over the spontaneous breathing trial (SBT) between patients who succeeded or failed the SBT.**

**Table S1. Numbers and proportions of spontaneous breathing trial (SBT) failure criteria identified in the 33 patients who failed the SBT.**

| **SBT failure criteria** | n (%) |
| --- | --- |
| Respiratory rate ≥ 35 breaths/min or increase ≥ 50% from baseline | 18 (55) |
| PaCO2 > 50 mmHg | 16 (48) |
| Systolic arterial pressure > 180 or < 90 mmHg | 16 (48) |
| SpO2 ≤ 90% or PaO2 ≤ 50 mmHg with FiO2 ≥ 50% | 9 (27) |
| Heart rate ≥ 140 bpm, de novo supraventricular or ventricular arrhythmia | 1 (3) |
| Alteration of consciousness | 0 (0) |

The total of failure criteria is greater than 33 because several criteria could be present in a same patient.

**Table S2. Respiratory distress assessment over the spontaneous breathing trial (SBT) between patients who succeeded or failed the SBT.**

| **Variables** | **Before** | **2-min** | **15-min** | **30-min (end)** | **P-value** |
| --- | --- | --- | --- | --- | --- |
| **Whole (n = 58)**  *f*R/VT, *breaths/min/L*  MV-RDOS ≥ 2.6, *n (%)*  **MV-RDOS value**  Heart rate, *beats/min*  Respiratory rate*, cycles/min*  Use of neck muscle during inspiration*, n (%)*  Abdominal paradox during inspiration*, n (%)*  Facial expression of fear*, n (%)* | 47 (34–66)  10 (17)  2.2 (2.0 2.3)  92 (84 100)  22 (17–27)  5 (9)  0 (0)  3 (5) | 64 (49–79)*  21 (38)  2.3 (2.2–4.2)*  96 (88–102)  25 (20–30)*  11 (19)  3 (5)  10 (17) | 67 (45–89)*  21 (53)  2.7 (2.2–4.3)*  96 (86–102)  25 (21–31)  12 (30)  5 (13)  5 (13) | 72 (49–107)*  22 (56)  3.9 (2.2–4.5)*  98 (89–105)  27 (22–32)*  13 (33)  6 (15)  8 (21) | <0.001  <0.001  <0.001  0.247  0.008  0.011  0.015  0.017 |
| **SBT Success (n = 25)**  *f*R/VT, *breaths/min/L*  MV-RDOS ≥ 2.6, *n (%)*  **MV-RDOS value**  Heart rate, *beats/min*  Respiratory rate*, cycles/min*  Use of neck muscle during inspiration*, n (%)*  Abdominal paradox during inspiration*, n (%)*  Facial expression of fear*, n (%)* | 37 (29–54)  0 (0)  2.1 (2.0–2.2)  91 (84–98)  19 (14–23)  0 (0)  0 (0)  0 (0) | 52 (41–72)  3 (12)  2.2 (2.1–2.4)  92 (86–102)  23 (20–26)  1 (4)  0 (0)  3 (12) | 50 (40–72)  8 (32)  2.3 (2.1–4.1)*  92 (82–101)  22 (18–26)  5 (20)  1 (4)  2 (8) | 56 (41–78)*  10 (40)  2.4 (2.1–4.2)*  93 (87–103)  24 (21–28)*  6 (24)  1 (4)  3 (12) | 0.014  0.002  0.011  0.782  0.061  0.015  1.000  0.363 |
| **SBT Failure (n = 33)**  *f*R/VT, *breaths/min/L*  MV-RDOS ≥ 2.6, *n (%)*  **MV-RDOS value**  Heart rate, *beats/min*  Respiratory rate*, cycles/min*  Use of neck muscle during inspiration*, n (%)*  Abdominal paradox during inspiration*, n (%)*  Facial expression of fear*, n (%)* | 56 (39–73)  10 (24)  2.3 (2.0–2.6)  94 (84–101)  22 (18–28)  5 (15)  0 (0)  3 (9) | 68 (57–88)  18 (55)  2.6 (2.2–4.5)  99 (90–104)  28 (24–32)  10 (30)  3 (9)  7 (21) | **n=15**  93 (60–141)  13 (87)  4.3 (2.8–5.3)  97 (88–109)  31 (27–34)*  7 (47)  4 (27)  3 (20) | **n=14**  94 (60–141)  12 (86)  4.8 (4.1–6.2)  101 (98–116)  33 (27–38)*  7 (50)  5 (36)  5 (36) | <0.001  <0.001  <0.001  0.122  0.006  0.041  <0.001  0.185 |

MV-RDOS, mechanical ventilation - respiratory distress observation scale; *f*R, respiratory rate; VT, expired tidal volume.

* Dunn’s multiple comparisons test with p<0.05, compared to Before
